# Tuning Curvature and Stability of Monoolein Bilayers by Designer Lipid-Like Peptide Surfactants

**DOI:** 10.1371/journal.pone.0000479

**Published:** 2007-05-30

**Authors:** Anan Yaghmur, Peter Laggner, Shuguang Zhang, Michael Rappolt

**Affiliations:** 1 Institute of Biophysics and Nanosystems Research (IBN), Austrian Academy of Sciences, Graz, Austria; 2 Center for Biomedical Engineering, Massachusetts Institute of Technology, Cambridge, Massachusetts, United States of America; University of East Piedmont, Italy

## Abstract

This study reports the effect of loading four different charged designer lipid-like short anionic and cationic peptide surfactants on the fully hydrated monoolein (MO)-based Pn3m phase (Q^224^). The studied peptide surfactants comprise seven amino acid residues, namely A_6_D, DA_6_, A_6_K, and KA_6_. D (aspartic acid) bears two negative charges, K (lysine) bears one positive charge, and A (alanine) constitutes the hydrophobic tail. To elucidate the impact of these peptide surfactants, the ternary MO/peptide/water system has been investigated using small-angle X-ray scattering (SAXS), within a certain range of peptide concentrations (*R*≤0.2) and temperatures (25 to 70°C). We demonstrate that the bilayer curvature and the stability are modulated by: i) the peptide/lipid molar ratio, ii) the peptide molecular structure (the degree of hydrophobicity, the type of the hydrophilic amino acid, and the headgroup location), and iii) the temperature. The anionic peptide surfactants, A_6_D and DA_6_, exhibit the strongest surface activity. At low peptide concentrations (*R* = 0.01), the Pn3m structure is still preserved, but its lattice increases due to the strong electrostatic repulsion between the negatively charged peptide molecules, which are incorporated into the interface. This means that the anionic peptides have the effect of enlarging the water channels and thus they serve to enhance the accommodation of positively charged water-soluble active molecules in the Pn3m phase. At higher peptide concentration (*R* = 0.10), the lipid bilayers are destabilized and the structural transition from the Pn3m to the inverted hexagonal phase (H_2_) is induced. For the cationic peptides, our study illustrates how even minor modifications, such as changing the location of the headgroup (A_6_K vs. KA_6_), affects significantly the peptide's effectiveness. Only KA_6_ displays a propensity to promote the formation of H_2_, which suggests that KA_6_ molecules have a higher degree of incorporation in the interface than those of A_6_K.

## Introduction

The physicochemical properties of the bicontinuous lipid-based cubic phases and their similarity to the cubic biomembranes found in living cells have received considerable attention [1]–[10]. These self-assembled structures are vital for several biological processes inside living cells such as for protein function and membrane fusion [1]–[7], [11]–[19]. Putton and Carey [20] observed for first time these cubic phases during their studies on fat digestion *in vitro*, and later these fascinating nanostructures were discovered in various other cells [1], [6]. The formation of cubic phases was also found in *E. coli* and *A. laidlawii* lipid extracts, which are rich in phosphatidylethanolamine and cardiolipin as well as monoglycosylglycerol [21]–[23]. Further, there is an increasing evidence that peptides (such as viral peptides) and proteins can induce the formation of non-lamellar structures (inverted type hexagonal and cubic phases) in biological cells [1]–[4], [7], [11]–[18].

Over the past few decades, many investigations have been carried out on the phase behavior of surfactant-like lipid/water systems [24]–[35]. Especially, several studies were reported on the temperature-water content phase diagrams of various binary monoglycerides/water systems [28]–[36]. Among these systems, the most studied monoolein (MO)/water binary mixture forms reverse isotropic micellar solution (L_2_), lamellar (L_α_), inverted type hexagonal (H_2_), and cubic (V_2_) liquid crystalline phases [26]–[34]. V_2_ is a three-dimensional (3-D) bicontinuous phase composed of bilayers [1], [8], [37]–[39], which separate two aqueous channel networks (the diameter of the fully swollen aqueous channel is about 40 Å). Considerable efforts have been invested also on dispersing these viscous bulk phases for the formation and the structural characterization of cubosomes and hexosomes [40]–[48].

**Figure 1:**
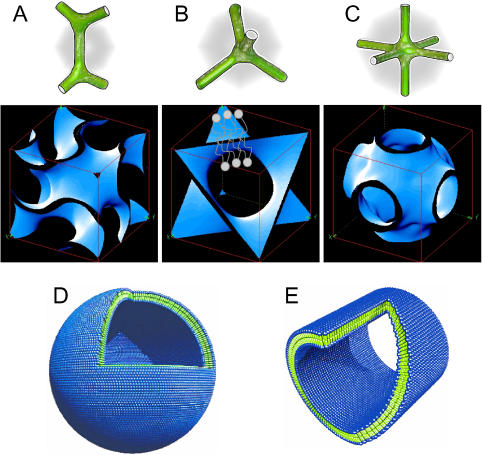
The bicontinuous gyroid (A), diamond (B) and primitive (C) cubic phases are shown. The bilayer mid-planes are displayed in blue and the corresponding skeletal graphs are presented on top. In parts (D) and (E), the proposed molecular models of peptide surfactant nanostructures in water are illustrated [66], [68], [70]: a peptide nanotube, and a peptide nanovesicle are presented, respectively. The average diameters of these self-assembled structures are about 300–500 Å. Color code: green for the hydrophobic tails; and blue for the charged headgroups. (The figures in panel A–C have been adapted with permission from reference [8] and the figures in D and E have been taken with permission from reference [68].)

The MO/water system displays two different types of bicontinuous cubic phases depending on the water content [28]–[34]: cubic assemblies with Ia3d (the gyroid type, C_G_) and Pn3m (diamond type, C_D_) symmetries, respectively. These cubic phases exhibit the lowest curvature inhomogeneity [1] and are complemented by the cubic phase with Im3m symmetry (the primitive type, C_P_), which is found in various other lipid systems [49], [50] and their aqueous dispersions [40]–[42], [50]. The water channel connectivity and the surface topology of these bicontinuous cubic phases are illustrated in Figure 1. MO-based bicontinuous cubic phases and their aqueous dispersions have been of great interest for various novel applications including for the crystallization of membrane proteins [51]–[54], and for the solubilization of different hydrophilic and lipophilic guest molecules such as vitamins, essential oils, and drugs [55]–[58]. In particular, these nanostructured systems are promising for the formation of effective drug delivery systems and have great potential applications in food, pharmaceutical, and cosmetic industry. The stability of such cubic phases depends on various parameters such as water content, temperature as well as on the type and the amount of the solubilized guest molecules [55]–[59]. For instance, the addition of lipophilic molecules [45], [60] leads to a structural transition from the V_2_ to H_2_, while amphiphilic molecules with a propensity of having positive spontaneous curvatures such as sodium oleate [60] lead to a transition from V_2_ to L_α_ (in literature, the sign of curvature is defined to be positive for surfaces of normal oil in water aggregates and negative for surfaces of inverse mesophases).

Moreover, the effect of various peptides on the stability of MO-based cubic phases has been subject of several studies [61]–[64]. It was found that the electrostatic interactions in the membranes incorporating negatively (or positively) charged lipids are controlling the structural lamellar-nonlamellar transitions, i.e. the nature of these guest peptides, the peptide-MO ratio, and salt concentration play an important role in modulating the self-assembled nanostructure.

In this work, we report on the effect of four different short lipid-like designer peptide surfactants on the fully hydrated Pn3m-phase of monoolein (MO). Our aim is to understand the key factors that tune the bilayer curvature and to gain further insight into the mechanism of the structural transformations induced by the addition of these molecules. We also checked the possibility to functionalize the bicontinuous cubic phase by the addition of small amount of peptide surfactant. It should be noted that the concept of *functionalization* [65] means to control the solubilization capacity of the liquid crystalline phases by the inclusion of specific anchors such as charged or long-chain amphiphilic molecules.

**Figure 2:**
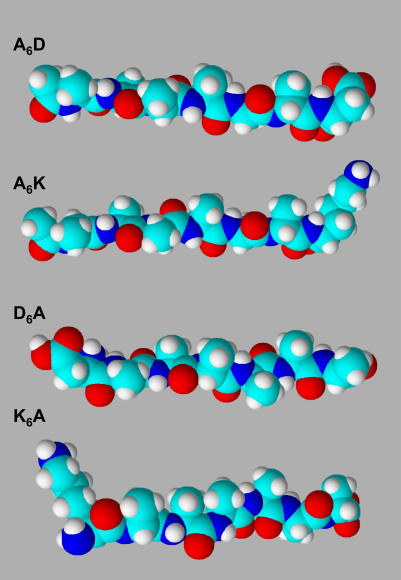
From top to bottom: the molecular models of the peptide surfactants A_6_D, A_6_K, DA_6_, and KA_6_. Aspartic acid (D) bears two negative charges, and lysine (K) bears one positive charge. Alanine (A) constitutes the hydrophobic tails. The peptides A_6_D and A_6_K were synthesized with the head group at the C-terminus. In the opposite arrangement, the peptides DA_6_ and KA_6_ were synthesized with the head group at the N-terminus. All used peptides are similar to biological phospholipids: each peptide is 25 Å in length. The color scheme as follows: carbon, cyan; nitrogen, blue; oxygen, red; and hydrogen, white.

The used peptides [66]–[72] are short cationic (A_6_K & KA_6_) and anionic (A_6_D & DA_6_) with an approximate length of 25 Å, which have been designed to mimic biological phospholipids (Figure 2). These peptides contain seven amino acid residues and are amphiphilic with a hydrophilic headgroup and a hydrophobic tail. For example, in the peptide surfactant A_6_D, the hydrophilic head has an aspartic acid (D) at the C-terminus, whereas the hydrophobic tail consists of six consecutive hydrophobic amine acids (alanine, A) with an acetylated N-terminus, eliminating the positive charge. We note that this 7-residue peptide has two negative charges at the C-terminus. Thus, these peptides exhibit self-assembling behavior akin to phospholipids with distinct critical aggregate concentration (CAC) values, also commonly referred to as critical micelle concentration (CMC) [69]. The CAC value [69], [71] corresponds to approximately 1.6 mM for A_6_D in pure water, and A_6_K has a CAC of ≈1.5 mM. Moreover, these designer peptide surfactants not only self assemble to form nanotubules and nanovesicles in water [66]–[68] with diameters of ∼300–500 Å (Figure 1D & E), but these “peptergents”, referring to peptides with detergent properties [72], represent a new class of biomaterials with excellent potential to solubilize, stabilize, and crystallize membrane proteins and enzymes [70]–[72].

The present study is organized in the following 4 sections: in the first two the effects of anionic and cationic peptides on the stability of Pn3m phase of the MO/water system are discussed. In section 3, the impact of headgroup location for the anionic A_6_D vs. DA_6_ and the cationic A_6_K vs. KA_6_ surfactants is described. In section 4, the phase behavior is examined on the basis of peptide concentration *versus* temperature diagrams. Finally, we propose hypotheses concerning the biological relevance and future applications.

## Results and Discussion

### Effect of anionic peptide surfactant

**Figure 3:**
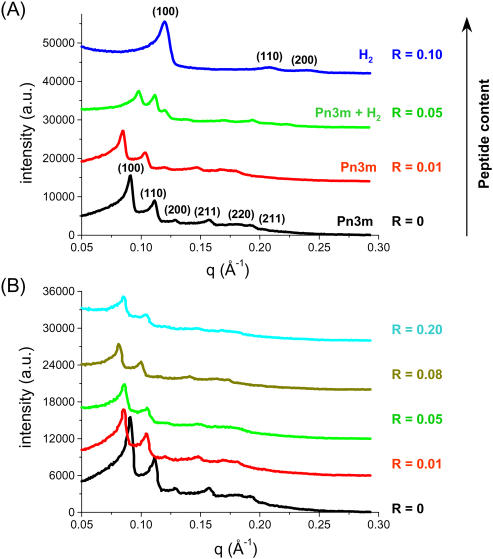
Impact of the anionic peptide A_6_D (A) and the cationic peptide A_6_K (B) on the MO-based fully hydrated Pn3m phase at 25°C. The experiments were carried out within a certain range of peptide concentrations (*R*≤0.2). The samples were formed in excess water at pH = 7.4 and contain a total amount of 18 wt% lipid (MO plus peptide). For better visibility the intensities are shifted by arbitrary constants.

The effect of anionic peptide surfactants on the stability of the MO-based Pn3m phase (Q^224^) has been examined by SAXS. In Figure 3A, the scattering curves for A_6_D-loaded MO/water phases with 4 different *R* values (in the range of 0 to 0.10) are shown at 25°C: in the absence of peptide (at *R* = 0), the diffraction pattern is indexed in accordance to a cubic Pn3m lattice. At low A_6_D concentration (at *R* = 0.01), the cubic Pn3m structure is preserved, but the peaks are shifted to lower *q* values, thus the corresponding lattice parameter, *a*, increases (Table 1). At *R* = 0.05, the diffraction pattern displays a coexistence of the former Pn3m cubic phase with a newly-formed H_2_ phase. A further increase in the peptide content (*R* = 0.1, which is the highest *R* value used for this peptide) leads to a complete structural transformation. The three observed peaks are identified by the (100), (110), and (200) reflections of the H_2_ phase. In short, we found that increasing the peptide concentration leads to structural transitions in the order V_2_ (Pn3m)→Pn3m & H_2_→H_2_. In addition, the diffraction peaks shift as a function of peptide concentration to higher *q* values, which mean a decrease in the lattice parameters.

**Table 1:** Unit cell parameter, *a*, of the MO-based systems at 25°C.

Investigated system	*T* (°C)	*R*	Phase	*a (Pn3m*) (Å)	*a (H_2_)* (Å)
MO-water	25	0	Pn3m	97.75	–
MO-A_6_D-water	25	0.01	Pn3m	104.19	–
	25	0.05	Pn3m & H_2_	89.65	64.41
	25	0.10	H_2_	–	60.02
MO-A_6_K-water	25	0.01	Pn3m	104.03	–
	25	0.05	Pn3m	104.03	–
	25	0.08	Pn3m	106.56	–
	25	0.10	Pn3m	109.26	–
	25	0.20	Pn3m	104.42	–
MO-DA_6_-water	25	0.006	Pn3m	98.90	–
	25	0.01	Pn3m	104.14	–
	25	0.05	Pn3m	106.53	–
	25	0.08	Pn3m	103.85	–
	25	0.10	H_2_	–	60.25
MO-KA_6_-water	25	0.01	Pn3m	104.53	–
	25	0.05	Pn3m	99.22	–
	25	0.10	Pn3m & H_2_	95.55	65.18
	25	0.20	traces of Pn3m & H_2_	undetected	63.86

The samples were formed at pH = 7.4 and contain 18 wt% lipid mixture (MO & peptide surfactant).

It is well known that the influence of guest molecules on phase transitions in liquid crystalline phases is closely related to the degree of their penetration in the membrane interface as well as to their ability to alter the spontaneous curvature of the monolayer leaflets [45], [56]–[60]. To shed some light on the impact of the designer peptides, we will particularly discuss the ‘effective’ molecular geometry of the membrane constituents and analyze its influence on forming diverse supramolecular structures. In a basic approach, the molecular shape can be described by the critical packing parameter (CPP) or the molecular wedge shape factor, which is defined as:
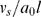
(1)where *v_s_* is the hydrophobic chain volume, *a_0_* is the headgroup area, and *l* is the hydrophobic chain length [73]. This packing parameter is controlled by various factors such as surfactant's molecular shape, temperature, hydration, the presence of hydrophilic or hydrophobic guest molecules, and electrostatic effects [16], [29]–[33], [35], [45]–[50], [61], [62].

**Figure 4:**
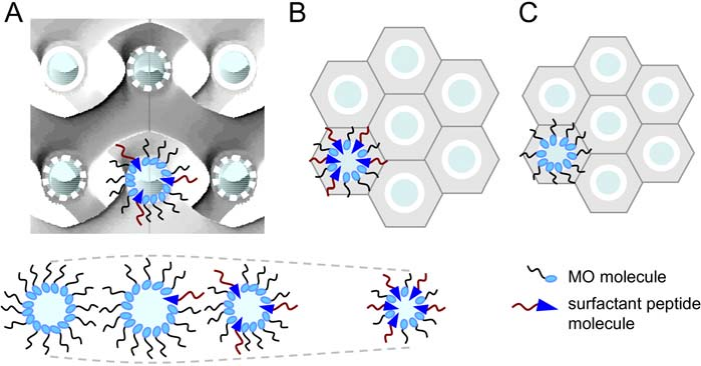
A schematic description of the influence of loading short designer peptide surfactants (cationic and anionic molecules) onto the fully hydrated Pn3m phase. (A) At low peptide content the Pn3m phase with its two interwoven water networks (marked by full and dashed circles) is preserved, (B) at high peptide content the H_2_ phase is induced and (C) for comparison the H_2_ phase is also depicted for the unloaded MO/water system. (The figure in panel A has been adapted with permission from the [ref. 34].)

In our study, at low A_6_D content (*R* = 0.01), the increase in the unit cell parameter, *a*, of the Pn3m phase (Table 1) is most probably attributed to the electrostatic repulsions between the negative charges of the peptides, which are incorporated into the electrically neutral MO-based membrane interface. The electrostatic forces will increase the distance of the negative charges and hence lead to an increase in the *a_0_* value at the water-lipid interface (see Figure 4). However, the electrostatic repulsions are not strong enough to increase significantly the CPP value or in other words to flatten the bilayer completely. The cubic phase Pn3m remains stable. In contrast, at higher peptide concentrations the lattice Pn3m lattice shrinks and at a certain peptide's concentration the structural transformation from Pn3m to H_2_ is observed. This can be attributed to an altered water solubilization in the self-assembled structure.

When the content of A_6_D is high, a considerable fraction of A_6_D molecules are incorporated into the MO matrix. As a consequence the amount of solubilized water at the lipid water interface decreases, i.e. CPP value increases due a reduction in *a_0_* and finally triggers the formation of the H_2_ phase. It was reported that the transformation from the Pn3m to the H_2_ structure [33] in MO-water at room temperature increases the CPP value from 1.31 to ∼1.70. However, the formation of the H_2_ phase is not only governed by a reduction in bending energy. The release of curvature frustration has a price, which is the build-up of packing frustration within the newly-formed hexagonal phase [74]. As seen in Figure 4, the closest packing of rods demands that each lipid cylinder has to fill out the space of a hexagon. This signifies that the lipid lengths must vary, i.e. lipids oriented towards the hexagon corners have a maximum length and those oriented perpendicular to the hexagon faces are the shortest. This variation in lipid length *l* does not depend on the water core radius, but can directly be determined from the lattice parameter of the H_2_ phase [75]:
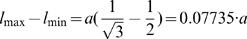
(2)It is important to note that the used designer peptides in this study (Figure 2) are about 7 Å longer then that of MO molecule, which at full hydration conditions has a length of about 18 Å [34], [76]. Therefore, it suggests that the designer peptides preferentially are localized in the corners of the hexagon and hence reduce the packing frustration within the hexagonal lattice. It is known that the interstitial regions [77] in H_2_ phase account for a volume fraction of about 9%. Our results show that the threshold concentration of the anionic peptides to induce the H_2_ phase is in the same order of magnitude (*R* = 0.05–0.1). Moreover, applying equation (2) to the A_6_D induced H_2_ phase with *a* ∼64 Å (*R* = 0.05, Table 1) results in a lipid length variation of 5 Å, which resembles just the difference in length of MO and peptide. A strong reduction of interstitial energy contributions would also explain in part the strongly reduced transition temperatures from Pn3m to H_2_. This transition occurs in the binary MO/water system at ∼90°C [32].

An additional interesting point is related to the peptide chains, which should have also an influence onto the spontaneous curvature of the MO monolayers. It is known that the hydrophobic tail packing for the surfactant-like peptides is quite different from that reported for conventional surfactants, most likely because of the intermolecular interactions due to the hydrogen bonds between peptide backbones [66]–[68]. Therefore, in pure peptide domains hydrophobic tails are rather tightly packed, leading to a local increase in the membrane rigidity. At this stage, however, we are not able to predict the overall rigidity of the composite MO/peptide leaflets and how rather rigid peptide chains influence the overall lateral chain pressure.

Interestingly, in aqueous medium our short designer peptide surfactants [68], [70] such as A_6_D favors the formation of bilayer structures with an estimated thickness of 50 Å (Figures 1D & 1E). Therefore, it is worthy noting that in our study the presence of bilayer-forming peptide in the MO/A_6_D mixture promotes the formation of H_2_ instead of the planar L_α_ (structure with zero curvature). Different findings were reported by Yamazaki and his coworkers [62] on the effect of the positively charged peptide WLFLLKKK, which was loaded with similar concentrations, onto the MO-based Pn3m phase. This peptide with a relatively large positively charged headgroup (KKK) induces the structural transformation from V_2_ (Pn3m) *via* V_2_ (Im3m symmetry) to L_α_. The reduction in bilayer curvature was explained in terms of increased electrostatic repulsions due to the partition of the charged peptide molecules in the MO interfacial film. Likewise, Chupin et al. [63] observed in a MO-based system that cubic to L_α_ phase transition can also be facilitated in the presence of a transmembrane peptide. However, transmembrane peptides are also known to induce non-lamellar structures when inserted into various phospholipid/water systems [17], [78]–[80]. For example, WALP and KALP (α-helical transmembrane peptides), which with 16 to 31 amino acids are significantly larger than our peptide surfactants, promote the L_α_→V_2_ and/or H_2_ structural transitions [17], [78], [79]. In particular, temperature and the peptide content dominate the lamellar/non-lamellar preference [17], [78]. Their propensity to form non-lamellar phases was rationalized by hydrophobic mismatch effects. When the hydrophobic thickness of the bilayer significantly exceeds the hydrophobic length of the transmembrane peptides [17], [78]–[80], a local disordering of the lipid acyl chains can occur, which serves as precursor for the formation of non-lamellar structures. However, in our study we are dealing with surfactant-like peptides and thus our results can not be explained within the framework of hydrophobic mismatch concept, but the transition from bicontinuous cubic to H_2_ phase is rather driven by the peptide structure and its amphiphilicity.

It comes clear that the impact of the designer peptide surfactants, which form well-ordered nanostructures in water (Figures 1D & 1E), is different from most short peptides reported in literature. In general, the previously studied peptides are not surfactant-like and thus do not form well-defined nanostructures in water. On the contrary, A_6_D must be classified as highly surface-active material, which can be exploited as a “tuneable inducer” of spontaneous membrane curvature.

### Effect of cationic peptide surfactant

Figure 3B shows the effect of the cationic peptide surfactant A_6_K on the stability of the fully hydrated MO-based Pn3m phase. SAXS patterns of the ternary MO/A_6_K/water system are displayed for various *R* values (in the range of 0 to 0.2) at 25°C. In the presence of A_6_K, all diffraction patterns are consistent with indexing a cubic lattice of the type Pn3m. This means that the structure is preserved after loading the cationic peptide surfactant. As shown in Figures 3A & 3B, similar behavior to the anionic A_6_D peptide is observed at low peptide content (at *R* = 0.01). In both systems, an increase of the unit cell parameter is observed, which is attributed to strong electrostatic repulsion between the positive charges of the peptide molecules incorporated into the interface (*R*≤0.1). However, a further increase of this cationic peptide concentration (*R* = 0.2) causes a counter effect, i.e. there is a slight decrease in the corresponding structure parameter of the Pn3m phase. Intriguingly, there is no indication for the formation of the H_2_ phase. This means that A_6_K is not as efficient as the negatively charged peptide A_6_D. It is plausible that the difference in their effectiveness is attributed to their degree of incorporation in the polar-apolar MO interface region. Both peptides are designed with a charged headgroup in the C-terminus to increase their solubility in water, using aspartic acid or lysine, respectively. However, our results suggest that A_6_K is only partially incorporated into the interface (a considerable part of it prefers to reside in the excess aqueous phase) and thus, most likely there is no significant reduction in the amount of solubilized water at the polar interface. Whereas, it seems that A_6_D prefers mainly to penetrate the membrane interface and thus is more efficient in inducing increasingly negative spontaneous monolayer curvature, which leads at a certain peptide concentration to the Pn3m→H_2_ transition.

### The impact of headgroup location on tuning the membrane curvature

**Figure 5:**
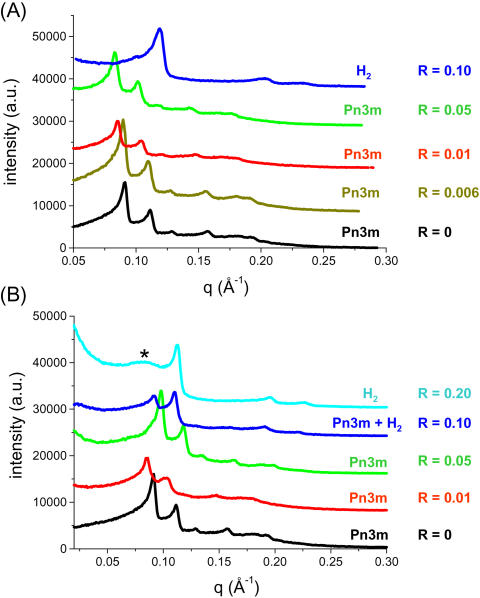
Impact of the anionic peptides DA_6_ (A) and KA_6_ (B) on the MO-based Pn3m phase at 25°C. *R* values range from 0–0.1. For better visibility the intensities were shifted by arbitrary constants. (The buffer and lipid concentration is the same as described in Figure 3.)

To investigate the role of the headgroup location and the importance of the headgroup charge, we compared the efficiency of the peptides A_6_D & A_6_K (the headgroup at the C-terminus, Figures 3A & 3B) with the peptide surfactants DA_6_ & KA_6_ (the headgroup at the N-terminus, Figure 5). It is well-known that a simple modification of the amino sequence of these designer peptide surfactants has a significant impact on their solubility in water [68]–[70]. For enhancing solubility, it is favorable for the peptides to have the headgroup at the C-terminus [68]–[70]. However, Zhang and his coworkers [68], [70] reported that the ordered nanostructures of these peptides in water are independent on the headgroup's location. Keeping this in mind, we checked the possible effect of slightly modified peptide structures on the MO-based Pn3m phase.

Figure 5A illustrates the impact of loading DA_6_ on the Pn3m structure. At low concentrations (*R* = 0.01 & 0.05), this peptide has a similar influence on the Pn3m phase as A_6_D does. Furthermore, it is also efficient at higher peptide concentrations in inducing the formation of H_2_ phase. Thus, the headgroup location plays only a minor role for the short anionic peptides. This means that our findings are similar to those reported on the aggregation behavior of the designer peptides in water [68], [70]. In contrast, the headgroup location does matter for the impact of cationic peptides. There are pronounced differences when A_6_K is replaced at the same peptide's concentration by KA_6_. As shown in Figure 5B, upon addition of KA_6_ the mean lattice parameter for the Pn3m phase increases as the *R* values changes in the range of 0–0.05. With a further increase in the peptide content (*R* = 0.1), the system exhibits a two-phase region of coexisting Pn3m and H_2_ structures, and at *R* = 0.20 a transition from Pn3m to H_2_ phase takes place. For this system, an additional broad peak appears at low q values (Figure 5B, _*_). This diffuse scattering contribution disappears when the sample is heated from 25 to 40°C (data not shown). Therefore, we believe that it arises from traces of a disordered bicontinuous diamond phase. The behavior of A_6_K (Figure 3B) contrasts sharply with that of KA_6_. A_6_K does not destabilize the MO-lipid bilayer. This can be attributed to the higher solubility [68] of A_6_K in the aqueous medium as compared to KA_6_, leading to a lower degree of incorporation in the MO bilayers and thus being less effective.

In brief, the positively charged KA_6_ has similar impact on the MO/water system as the negative peptides A_6_D & DA_6_, but it is less effective. Thus, the hydrophilicity of the headgroup seems to play an important role on tuning the membrane curvatures: replacing D with the less hydrophilic K decreases significantly the peptide's effectiveness. Figure 4 summarizes the main findings. Low peptide concentrations increase slightly the interface curvature due to the electrostatic repulsion at the headgroups, while at higher concentrations especially the anionic designer peptides induce a nanostructure transition from Pn3m to H_2_ due to a decrease in the amount of solubilized water in the interface region, and secondly to a release of the curvature frustration (compare with the discussion on anionic peptide surfactants).

In Figures 3 & 5, it is also worthy noting that all peptide-loaded samples display an increased diffuse scattering at low *q* values. An example for such effect is shown in Figure 5B. If we take into consideration that these surfactant peptides undergo self-assembly [65]–[70] in water and form nanovesicles and nanotubes (Figures 1D & 1E), this scattering contribution might be attributed to the formation of small peptide surfactant aggregates in co-existence with the ternary MO/peptide/water phases.

### Effect of temperature on MO/peptide/water systems

**Figure 6:**
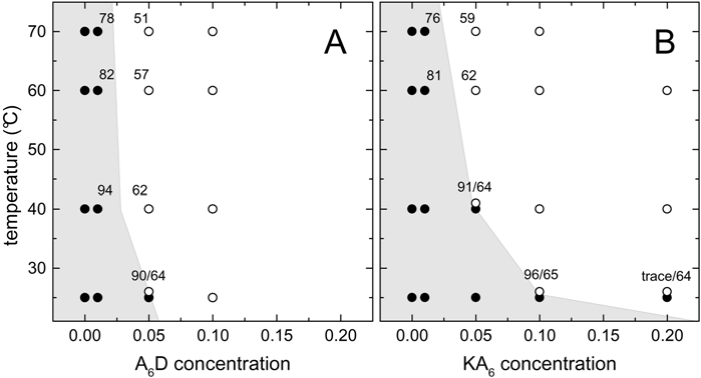
Phase diagrams of MO-based systems with differing A_6_D (A) and KA_6_ content (B), respectively. The structural investigations were carried out for *R* values in the range of 0–0.2. The Pn3m phase regions in these diagrams have been highlighted in light grey and approximate phase boundaries are depicted. The lattice parameter values, *a*, for some of the Pn3m (•) and H_2_ (O) phases are given in Ångstrøms.

As we have discussed in the previous sections, the formation of H_2_ phase is promoted most efficiently by the anionic peptides. In contrast, the addition of cationic peptides to the MO/water system displays a reduced efficiency in varying the monolayer curvature. In Figure 6A, we compare the temperature-peptide concentration diagram of MO/A_6_D/water with that of MO/KA_6_/water (Figure 6B). For better distinction, the cubic phase regions in these diagrams have been highlighted in light grey. It is worth noting that the phase boundaries are only very roughly estimated and do not allow any deduction of phase transition temperatures or phase transition concentrations, respectively. It is clear though that especially in the low temperature regime A_6_D is more efficient than KA_6_ in destabilizing the membrane bilayer. However, rising temperature increases the CPP value and therefore enhances the spontaneous negative curvature by two ways: it reduces the value of *a_0_* due to the headgroup's dehydration and it enhances simultaneously the value of *v_s_*. Thus, in this scenario the bilayer thickness as well as the water core radii decrease monotonously with temperature. At higher temperatures, our results for both ternary systems (Figure 6) indicate that the differences in peptide efficiency are not that pronounced any more. Here, the hydrophobic chain pressure [81] becomes the dominating driving force and hence gains importance in tuning membrane curvature. In other words, a further change in the interfacial area due to headgroup dehydration seems to play a less significant role at higher temperatures.

**Figure 7:**
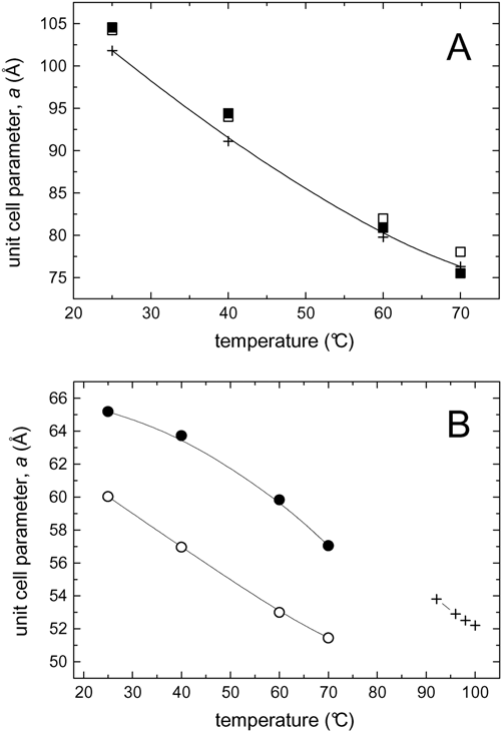
(A) Variation of the unit cell parameter, *a*, during heating for the fully hydrated Pn3m phase of the binary MO/water mixture [26] (+), and for the peptide-loaded ternary systems at *R* = 0.01: MO/A_6_D/water (□), and MO/KA_6_/water (▪). (B) Variation of *a(T)* for the H_2_ phase of the binary MO/water mixture (+) [26], and for the peptide-loaded ternary systems at *R* = 0.1: MO/A_6_D/water (O), and MO/KA_6_/water (•).

In Figure 7A, we take a closer look to the temperature behavior of the Pn3m phase at *R* = 0.01. Our results reveal that heating the A_6_D- and KA_6_-loaded systems display a very similar trend of behavior when compared to the unloaded binary MO/water system [32]. As shown also in Table 2, the decline of the lattice parameter with temperature is nearly identical in all cases. The interpretation is straightforward: the CPP value increases with temperature and hence the spontaneous curvature at the interface becomes increasingly negative. This holds true also for the H_2_ phase, but the situation is more complex: it is important to recall that the H_2_ phase in the binary MO/water system [27], [32] exists only within a small regime at high temperatures (∼90–100°C). Further, increasing temperature from 90 to 100°C causes a slight decrease in lattice parameter of this mesophase from 54 to 52 Å (Figure 7B, +). At even higher temperatures (>100°C), a fluid isotropic fluid (inverted micellar solution, L_2_) is formed.

**Table 2:** Unit cell parameter, *a*, of the ternary MO/A_6_D/water and MO/KA_6_/water systems.

Investigated system	*T* (°C)	*R*	Phase	*a (Pn3m*)(Å)	*a (H_2_)*(Å)
MO-A_6_D-water	25	0.01	Pn3m	104.19	–
	40	0.01	Pn3m	93.96	–
	60	0.01	Pn3m	81.97	–
	70	0.01	Pn3m	78.04	–
	25	0.05	Pn3m & H_2_	89.65	64.41
	40	0.05	H_2_	–	61.57
	60	0.05	H_2_	–	57.28
	70	0.05	H_2_	–	55.17
	25	0.10	H_2_	–	60.02
	40	0.10	H_2_	–	56.95
	60	0.10	H_2_	–	52.99
	70	0.10	H_2_	–	51.44
MO-KA_6_-water	25	0.01	Pn3m	104.53	–
	40	0.01	Pn3m	94.39	–
	60	0.01	Pn3m	80.88	–
	70	0.01	Pn3m	75.55	–
	25	0.05	Pn3m	99.22	–
	40	0.05	Pn3m & H_2_	90.52	64.33
	60	0.05	H_2_	–	61.61
	70	0.05	H_2_	–	59.33
	25	0.10	Pn3m & H_2_	95.55	65.18
	40	0.10	H_2_	–	63.72
	60	0.10	H_2_	–	59.82
	70	0.10	H_2_	–	57.04
	25	0.20	traces of Pn3m & H_2_	undetected	63.86
	40	0.20	H_2_	–	60.95
	60	0.20	H_2_	–	57.06

The experiments were carried out at four different temperatures in the range of 25–70°C. The samples were formed at pH = 7.4 and contain 18 wt% lipid mixture (MO & peptide surfactant).

As can be judged from Figure 7B, there is a significant impact of the studied peptides on the behavior of the H_2_ phases when compared to that of the binary system: firstly, there is a strong reduction in the Pn3m→H_2_ transition temperatures. At *R* = 0.05, loading A_6_D and KA_6_ reduces the transition temperature from approximately 90 to 25, and 40°C, respectively (Figure 6). Secondly, the H_2_ region in the phase diagram is drastically increased. Thirdly, the addition of the designer peptides enlarges the diameter of the formed hydrophilic cylinders (Tables 1 & 2 and Figure 7B). For instance at *R* = 0.1, for the KA_6_-loaded system the smallest lattice parameter measured has a value of 58 Å (Figure 7B, •). However, an interpolation of the *a(T)* plot would lead to similar lattice parameter values as recorded for the binary MO/water system at 90–100°C. The behavior is different, when the most efficient peptide A_6_D is loaded (Figure 7B, O). While *a(T)* decreases with approximately −0.2 Å/°C, which is almost the same as for the binary MO/water system, the *a* value drops down to 52 Å already at 70°C (compare O with + in Figure 7B). This underlines once more the exceptional strong ability of the designer peptide A_6_D to reduce the effective interface area per molecule, since an increased chain pressure alone can not cause such high curvatures between 60 and 70°C.

### Relevance of the short designer peptide surfactants

The self-assembly of biological molecules for the design novel materials with well-defined nanostructures is increasingly exploited in biotechnology [70]. For instance, the designer peptide surfactants of this work undergo self-assembly and form well-defined systems useful for various potential applications such as the encapsulation, the solubilization, or the crystallization of active biomolecules [66]–[72]. Here, we have shown that these peptide surfactants can also be used to stabilize different non-lamellar mesophases, which have also biological relevance. For instance, Bechinger and Lohner [82] pointed out that peptide surfactants, which are present in plant and human cells, modulate the antimicrobial activities of biomembranes.

The short designer peptide surfactants are tuneable nanobiomaterials. It is easy to modify the peptide hydrophobic tail as well as its headgroup. For instance, the degree of hydrophobicity can be fine-tuned by replacing alanine (A) with more hydrophobic amino acids such as valine (V), or leucine (L). Furthermore, the degree of hydrophilicity can be varied by increasing the number of the hydrophilic amino acids on the peptide's backbone and by replacing the negatively charged aspartic acid or the positively charged lysine by other hydrophilic amino acids such as the negatively charged glutamic acid (E) or the positively charged histidine (H) or arginine (R).

As a final point we would like to show up a practical route for functionalizing bicontinuous cubic phases by the addition of peptide surfactants. We anticipate that the inclusion of small amount of the charged short designer surfactants A_6_D (or A_6_K) into the MO interfacial film will enhance the loading capacity of positively (or negatively) charged water-soluble active molecules in the hydrophilic channels of the Pn3m phase. Enhancement of solubilization capacity of active guest molecules is an important issue in various pharmaceutical, food, and cosmetic applications. In particular, today the formation of suitable carriers for loading charged drugs or peptides is of great interest.

### Conclusions

The present work focuses on the influences of short charged designer peptides, which mimic the properties of anionic and cationic surfactant molecules, on the fully hydrated Pn3m nanostructure of MO. The following major conclusions can be drawn from our investigations:

1. **Charged designer peptide surfactants:** the structure of the bicontinuous cubic phases can be modulated by adding short negatively or positively charged peptide surfactants. First, they allow to tune the water channel size of the crystalline phases at low peptide's content, and second, the transition of the nanostructure from Pn3m to H_2_ can be induced either by augmenting the peptides' concentration at room temperature or by increasing the temperature at a fixed peptide concentration. We found that the effectiveness of these peptide surfactants depends on the headgroup location and its structure (the type of the hydrophilic amino acid).
2. **Model systems as tools for studying biological systems:** lipids (such as monoglycerides and phospholipids) form in water various mesophases (lamellar, hexagonal or cubic structures). The nanostructures of these mesophases depend on temperature, water content and the molecular structure of the lipids (single or double chained, saturation degree, and length of fatty acyl chain). Our present lipid/peptide surfactant membrane model provides the basis to learn more about possible effects of active molecules in biomembranes and helps to understand structural transitions that occur within biological cells.
3. **Potential applications:** *functionalization* of bicontinuous cubic phases is possible by the addition of charged short peptides. These serve as anchors in the water/lipid interface and allow enhancing the loading capacity of charged active molecules.

## Materials and Methods

### Materials

Monoolein (1-monooleoyl-rac-glycerol, MO, purity: 99%) was purchased from Sigma Chemical Co. (St. Louis, Missouri, USA). Chloroform (CHCl_3_, purity: >99%) and 2,2,2-Trifluroethanol (TFE, purity: 99.8%) were supplied by Carl Roth GmbH (Karlsruhe, Germany). The used buffer was PBS (phosphate buffered saline contains 20 mM NaPi and 130 mM NaCl, pH 7.4). All ingredients were used without further purification.

### Peptide Surfactants: Design and Synthesis

The lipid-like peptide surfactants were synthesized by CPC Scientific (San Jose, CA) and characterized by the Biopolymers Laboratory at MIT (MA, USA). The design is based on creating a peptide of approximately phospholipid size containing a hydrophilic head and a hydrophobic tail [66]–[69]. The synthesis of these surfactant peptides are described in previous reports of Zhang and his coworkers [66]–[68]. The four peptides studied are A_6_K, A_6_D, KA_6_, and DA_6_. The first two are synthesized with the headgroup at C-terminus, whereas the last two with the headgroup at the *free* N-terminus (K = lysine, A = alanine, D = aspartic acid).

### Preparation of MO/Peptide Surfactant Samples

Lipid-like peptide surfactants with appropriate concentrations were dissolved in TFE while MO was dissolved in chloroform. These solvents were mixed and then evaporated using a gentle stream of nitrogen, followed by drying under vacuum for at least 12 hours in order to remove completely the residual organic solvent. The dry lipid-peptide film was hydrated by adding the PBS buffer and carrying out at least 5 freeze-thaw cycles between liquid nitrogen and room temperature and then homogenizing several times during the thawing steps by vigorous vortexing. The fully hydrated samples coexist with excess water and were formed with a fixed total lipid concentration of ∼18 wt%. In our study, we investigated the effect of varying the molar ratio *R* (*R* = moles of surfactant peptide/mole of MO) on the stability of the MO-based Pn3m phase. The prepared samples were incubated at room temperature for two to three weeks before carrying out SAXS measurements.

### X-Ray Scattering Measurements

The X-ray measurements were carried out on a small- and wide angle X-ray scattering camera with Kratky collimation [83] (SWAXS, System3, Hecus X-ray Systems, Graz, Austria) using a 4 kW rotating Cu-anode X-ray generator (Rigaku-Denki, MA, USA). The system incorporates a pulse-height discriminator, which is used in combination with a 10 µm Ni foil to obtain Cu K_α_ radiation (λ = 1.542 Å). Further, the camera is equipped with a Peltier-controlled variable-temperature cuvette (temperature resolution 0.1°C) and a linear one-dimensional position-sensitive detector (PSD 50-M, Hecus X-ray Systems, Graz, Austria) covering the *q*-range of 0.004 to 0.5 Å^−1^ (*q* = *2*π *sin*θ */*λ ). The system allows automatic serial exposures by a programmable temperature unit and time frame generator. The temperature scans were performed in heating direction. After equilibration of the samples for at least 600 s (waiting time) at the respective temperature, the SAXS pattern were recorded with exposure times of 1000 s. For indexing the different mesophases and calculating the corresponding unit lattice parameter *a*, we applied the respective reflection laws for the cubic and the hexagonal phases [8].

## References

[1] Hyde S, Andersson S, Larsson K, Blum Z, Landh T (1997). The language of shape. The role of curvature in condensed matter: physics, chemistry, biology.

[2] Luzzati V (1997). Biological significance of lipid polymorphism: the cubic phases.. Curr Opin Struct Biol.

[3] Lindblom G, Rilfors L (1989). Cubic phases and isotropic structures formed by membrane lipids - possible biological relevance.. Biochim Biophys Acta.

[4] de Kruijff B (1997). Lipids beyond the bilayer.. Nature.

[5] Garab G, Lohner K, Laggner P, Farkas T (2000). Self-regulation of the lipid content of membranes by non-bilayer lipids: a hypothesis.. Trends Plants Sci.

[6] Almsherqi ZA, Kohlwein SD, Deng Y (2006). Cubic membranes: a legend beyond the flatland of cell membrane organization.. J Cell Biol.

[7] Deng Y, Kohlwein SD, Mannella CA (2002). Fasting induces cyanide-resistant respiration and oxidative stress in the amoeba Chaos carolinensis: implications for the cubic structural transition in mitochondrial membranes.. Protoplasma.

[8] Rappolt M, Leitmannova A (2006). The biologically relevant lipid mesophases as “seen” by X-rays.. Planar lipid bilayers and liposomes.

[9] Seddon JM (1996). Lyotropic phase behaviour of biological amphiphiles.. Ber Bunsenges Phys Chem.

[10] Lewis RNAH, Mannock DA, McElhaney RN, Epand RM (1998). Membrane lipid molecular structure and polymorphism.. Lipid polymorphism and membrane properties. Current topics in membrane.

[11] Ellens H, Siegel DP, Alford D, Yeagle PL, Boni L (1989). Membrane fusion and inverted phases.. Biochemistry.

[12] Basáñez G, Gõni FM, Alonso A (1998). Effect of single chain lipids on phospholipase c-promoted vesicle fusion. A test for the stalk hypothesis of membrane fusion.. Biochemistry.

[13] Colotto A, Epand RM (1997). Structural study of the relationship between the rate of membrane fusion and the ability of the fusion peptide of influenza virus to perturb bilayers.. Biochemistry.

[14] Colotto A, Martin I, Ruysschaert J-M, Sen A, Hui SW (1996). Structural study of the interaction between the SIV fusion peptide and model membranes.. Biochemistry.

[15] Epand RM, Epand RM (1998). Modulation of lipid polymorphism by peptides.. Lipid polymorphism and membrane properties. Current topics in membrane.

[16] Li SJ, Yamashita Y, Yamazaki M (2001). Effect of electrostatic interactions on phase stability of cubic phases of membranes of monoolein/dioleoylphosphatidic acid mixtures.. Biophys J.

[17] Siegel DP, Cherezov V, Greathouse DV, Koeppe RE, Killian JA (2006). Transmembrane peptides stabilize inverted cubic phases in a biphasic length-dependent manner: implications for protein-induced membrane fusion.. Biophys J.

[18] Siegel DP (1999). The modified stalk mechanism of lamellar/inverted phase transitions and its implications for membrane fusion.. Biophys J.

[19] Simidjiev I, Stoylova S, Amenitsch H, Jávorfi T, Mustárdy L (2000). Self-assembly of large, ordered lamellae from non-bilayer lipids and integral membrane proteins *in vitro*.. PNAS.

[20] Patton JS, Carey MC (1979). Watching fat digestion.. Science.

[21] Andersson A-S, Rilfors L, Orädd G, Lindblom G (1998). Total lipids with short and long acyl chains from *Acholeplasma* form nonlamellar phases.. Biophys J.

[22] Wieslander Å, Nordström S, Dahlqvist A, Rilfors L, Lindblom G (1995). Membrane lipid composition and cell size of *Acholeplasma laidlawii* strain A are strongly influenced by lipid acyl chain length.. Eur J Biochem.

[23] Staudegger E, Prenner EJ, Kriechbaum M, Degovics G, Lewis RNAH (2000). X-ray studies on the interaction of the antimicrobial peptide gramicidin S with microbial lipid extracts: evidence for cubic phase formation.. Biochim Biophys Acta.

[24] Lindblom G, Larsson K, Johansson L, Fontell K, Forsén S (1979). The cubic phase of monoglyceride-water systems. Arguments for a structure based upon lamellar bilayer units.. J Am Chem Soc.

[25] Nilsson A, Holmgren A, Lindblom G (1994). An FTIR study of the hydration and molecular ordering at phase transitions in the monooleoylglycerol/water system.. Chem Phys Lipids.

[26] Chernik GG (2000). Phase studies of surfactant-water systems.. Curr Opin Colloid Interface Sci.

[27] Larsson K (1983). Two cubic phases in monoolein/water system.. Nature.

[28] Lutton ES (1965). Phase behavior of aqueous systems of monoglycerides.. JAOCS.

[29] Qiu H, Caffrey M (2000). The phase diagram of the monoolein/water system: metastability and equilibrium aspects.. Biomaterials.

[30] Qiu H, Caffrey M (1998). Lyotropic and thermotropic phase behavior of hydrated monoacylglycerols: Structure characterization of monovaccenin.. J Phys Chem B.

[31] Qiu H, Caffrey M (1999). Phase behavior of the monoerucin/water system.. Chem Phys Lipids.

[32] Briggs J, Chung H, Caffrey M (1996). The temperature-composition phase diagram and mesophase structure characterization of the monoolein/water system.. J Phys II France.

[33] Larsson K (1989). Cubic lipid-water phases: structures and biomembrane aspects.. J Phys Chem.

[34] Rappolt M, Di Gregorio GM, Almgren M, Amenitsch H, Pabst G (2006). Non-equilibrium formation of the cubic Pn3m phase in a monoolein/water system.. Europhy Lett.

[35] de Campo L, Yaghmur A, Sagalowicz L, Leser ME, Watzke H (2004). Reversible phase transitions in emulsified nanostructured lipid systems.. Langmuir.

[36] Misquitta Y, Caffrey M (2001). Rational design of lipid molecular structure: a case study involving the C19:1c10 monoacylglycerol.. Biophys J.

[37] Hyde ST (1996). Bicontinuous structure in lyotropic liquid crystals and crystalline hyperbolic surfaces.. Curr Opin Solid State Mater Sci.

[38] Chung H, Caffrey M (1994). The curvature elastic-energy function of the lipid/water cubic mesophase.. Nature.

[39] Hyde ST, Holmberg K (2001). Identification of lyotropic liquid crystalline mesophases.. Handbook of applied surface and colloid chemistry.

[40] Gustafsson J, Ljusberg-Wahren H, Almgren M, Larsson K (1996). Cubic lipid-water phase dispersed into submicron particles.. Langmuir.

[41] Gustafsson J, Ljusberg-Wahren H, Almgren M, Larsson K (1997). Submicron particles of reversed lipid phases in water stabilized by a nonionic amphiphilic polymer.. Langmuir.

[42] Larsson K (2000). Aqueous dispersions of cubic lipid-water phases.. Curr Opin Colloid Interface Sci.

[43] Spicer PT, Schwarz JA, Contescu C, Putyera K (2004). Cubosomes: bicontinuous liquid crystalline nanoparticles.. Dekker encyclopaedia of nanoscience and nanotechnology.

[44] Barauskas J, Johnsson M, Joabsson F, Tiberg F (2005). Cubic phase nanoparticles (cubosome): principles for controlling size, structure, and stability.. Langmuir.

[45] Yaghmur A, de Campo L, Sagalowicz L, Leser ME, Glatter O (2005). Emulsified microemulsions and oil-containing liquid crystalline phases.. Langmuir.

[46] Yaghmur A, de Campo L, Salentinig S, Sagalowicz L, Leser ME (2006). Oil-loaded monolinolein-based particles with confined inverse discontinuous cubic structure (*Fd*3*m*).. Langmuir.

[47] Angelova A, Angelov B, Papahadjopoulos-Sternberg B, Bourgaux C, Couvreur P (2005). Protein driven patterning of self-assembled cubosomic nanostructures: long oriented nanoridges.. J Phys Chem B.

[48] Sagalowicz L, Michel M, Adrian M, Frossard P, Rouvet M (2006). A Cryo-TEM study of the crystallographic structure and morphology of dispersed monoglyceride self-assembly structures.. J Microscopy.

[49] Landh T (1994). Phase behavior in the system pine needle oil monoglycerides-Poloxamer 407-water at 20.degree.. J Phys Chem.

[50] Yaghmur A, de Campo L, Sagalowicz L, Leser ME, Glatter O (2006). Control of the internal structure of MLO-based Isasomes by the addition of diglycerol monooleate and soybean phosphatidylcholine.. Langmuir.

[51] Rummel G, Hardmeyer A, Widmer C, Chiu ML, Nollert P (1998). Lipidic cubic phases: new matrices for the three-dimensional crystallization of membrane proteins.. J Struct Biol.

[52] Landau EM, Rummel G, Rosenbusch JP, Cowan-Jacob SW (1997). Crystallization of a polar protein and small molecules from the aqueous compartment of lipidic cubic phases.. J Phys Chem B.

[53] Caffrey M (2000). A lipid's eye view of membrane protein crystallization in mesophases.. Curr Opin Struct Biol.

[54] Caffrey M (2003). Membrane protein crystallization.. J Struct Biol.

[55] Drummond C, Fong C (2000). Surfactant self-assembly objects as novel drug delivery vehicles.. Curr Opin Colloid Interface Sci.

[56] Caboi F, Amico GS, Pitzalis P, Monduzzi M, Nylander T (2001). Addition of hydrophilic and lipophilic compounds of biological relevance to the monoolein/water system. I. Phase behavior.. Chem Phys Lipids.

[57] Caboi F, Nylander T, Razumas V, Talaikyte Z, Monduzzi M (1997). Structural effects, mobility, and redox behavior of vitamin K-1 hosted in the monoolein/water liquid crystalline phases.. Langmuir.

[58] Caboi F, Murgia S, Monduzzi M, Lazzari P (2002). NMR investigation on *Melaleuca Alternifolia* essential oil dispersed in the monoolein aqueous system: phase behavior and dynamics.. Langmuir.

[59] Borne J, Nylander T, Khan A (2000). Microscopy, SAXD, and NMR studies of phase behavior of the monoolein-diolein-water system.. Langmuir.

[60] Borne J, Nylander T, Khan A (2001). Phase behavior and aggregate formation for the aqueous monoolein system mixed with sodium oleate and oleic acid.. Langmuir.

[61] Masum SMD, Li SJ, Tarek SA, Yamazaki M (2005). Effect of positively charged short peptides on stability of cubic phases of monoolein/dioleoylphosphatidic acid mixtures.. Langmuir.

[62] Masum SMD, Li SJ, Tamba Y, Yamashita Y, Tanaka T (2003). Effect of de Novo designed peptides interacting with the lipid-membrane interface on the stability of the cubic phases of the monoolein membrane.. Langmuir.

[63] Chupin V, Killian JA, de Kruijff B (2003). Effect of phospholipids and a transmembrane peptide on the stability of the cubic phase of monoolein: implication for protein crystallization from a cubic.. Phase Biophys J.

[64] Kamo T, Nakano M, Kuroda Y, Handa T (2006). Effects of an amphipathic alpha-helical peptide on lateral pressure and water penetration in phosphatidylcholine and monoolein mixed membranes.. J Phys Chem B.

[65] Lynch ML, Ofori-Boateng A, Hippe A, Kochva K, Spicer PT (2003). Enhanced loading of water-soluble actives into bicontinuous cubic phase liquid crystals using cationic surfactants.. J Colloid Interface Sci.

[66] Vauthey S, Santoso S, Gong H, Watson N, Zhang S (2002). Molecular self-assembly of surfactant-like peptides to form nanotubes and nanovesicles.. Proc Natl Acad Sci U S A.

[67] Santoso S, Hwang W, Hartman H, Zhang S (2002). Self-assembly of surfactant-like peptides with variable glycine tails to form nanotubes and nanovesicles.. Nano Lett.

[68] von Maltzahn G, Vauthey S, Santoso S, Zhang S (2003). Positively charged surfactant-like peptides self-assemble into nanostructures.. Langmuir.

[69] Yang SJ, Zhang S (2006). Self-assembling behavior of designer lipid-like peptides.. Supramol Chem.

[70] Zhao X, Zhang S (2006). Molecular designer self-assembling peptides.. Chem Soc Rev.

[71] Zhao X, Nagai Y, Reeves PJ, Kiley P, Khorana HG (2006). Designer short peptide surfactants stabilize G protein-coupled receptor bovine rhodopsin.. Proc Natl Acad Sci U S A.

[72] Yeh JI, Du S, Tortajada A, Paulo J, Zhang S (2005). Peptergents: Peptide detergents that improve stability and functionality of a membrane protein, glycerol-3-phosphate dehydrogenase.. Biochemistry.

[73] Isrealachvilli JN, Mitchell DJ, Ninham BW (1976). Theory of self-assembly of hydrocarbon amphiphiles into micelles and bilayers.. J Chem Soc Faraday Trans II.

[74] Kozlov MM, Leikin S, Rand RP (1994). Bending, hydration and interstitial energies quantitatively account for the hexagonal-lamellar-hexagonal reentrant phase transition in dioleoylphosphatidylethanolamine.. Biophys J.

[75] Tate MW, Gruner SM (1989). Temperature dependence of the structural dimensions of the inverted hexagonal (HII) phase of phosphatidylethanolamine-containing membranes.. Biochemistry.

[76] Harper PE, Gruner SM (2000). Electron density modeling and reconstruction of infinite periodic minimal surfaces (IPMS) based phases in lipid-water systems. I. Modeling IPMS-based phases.. Eur Phys J.

[77] Duesing PM, Templer RH, Seddon JM (1997). Quantifying packing frustration energy in inverse lyotropic mesophases.. Langmuir.

[78] Killian JA, Nyholm TKM (2006). Peptides in lipid bilayers: the power of simple models.. Curr Opin Struct Biol.

[79] Morein S, Koeppe RE, Lindblom G, de Kruijff B, Killian JA (2000). The effect of peptide/lipid hydrophobic mismatch on the phase behavior of model membranes mimicking the lipid composition in *Escherichia coli* membranes.. Biophys J.

[80] Liu F, Lewis RNAH, Hodges RS, McElhaney RN (2001). Differential scanning calorimetric and ^31^P NMR spectroscopic study of the effect of transmembrane α-helical peptides on the lamellar-reversed hexagonal phase transition of phosphatidylethanolamine model membranes.. Biochemistry.

[81] Shearman GC, Ces O, Templer RH, Seddon JM (2006). Inverse lyotropic phases of lipids and membrane curvature.. J Phys Condens Matter.

[82] Bechinger B, Lohner K (2006). Detergent-like actions of linear amphipathic cationic antimicrobial peptides.. Biochim Biophys Acta.

[83] Laggner P, Mio H (1992). SWAX - a dual-detector camera for simultaneous small- and wide-angle X-ray diffraction in polymer and liquid crystal research. Nucl. Instrum.. Methods Phys Res.

